# Prognostic impact of immune microenvironment in laryngeal and pharyngeal squamous cell carcinoma: Immune cell subtypes, immuno-suppressive pathways and clinicopathologic characteristics

**DOI:** 10.18632/oncotarget.14242

**Published:** 2016-12-27

**Authors:** Georgia Karpathiou, Francois Casteillo, Jean-Baptiste Giroult, Fabien Forest, Pierre Fournel, Alessandra Monaya, Marios Froudarakis, Jean Marc Dumollard, Jean Michel Prades, Michel Peoc'h

**Affiliations:** ^1^ Department of Pathology, North Hospital, University Hospital of St-Etienne, St-Etienne, France; ^2^ Department of Head and Neck Surgery, North Hospital, University Hospital of St-Etienne, St-Etienne, France; ^3^ Department of Pneumonology, North Hospital, University Hospital of St-Etienne, St-Etienne, France; ^4^ Oncology Institute Lucien Neuwirth, St-Etienne, France

**Keywords:** PD-L1, CTLA4, macrophages, dendritic cells

## Abstract

**Background:**

Immune system affects prognosis of various malignancies. Anti-immune pathways like PD-L1 and CTLA4 are used by the tumor to overcome immune system and they serve as immunotherapy targets. The immune microenvironment of head-and-neck squamous cell carcinoma (SCCHN) has not been sufficiently studied.

**Patients and Methods:**

152 SCCHN were immunohistochemically studied for the expression of CD3, CD8, CD57, CD4, granzyme b, CD20, CD163, S100, PD-L1, CTLA4 and CXCR4.

**Results:**

CD3, CD8, CD57 and stromal S100 higher density is a good prognostic factor (p=0.02, 0.01, 0.02, 0.03 respectively). CTLA4 tumor expression is a poor prognostic factor (p=0.05). The rest immune cells do not affect prognosis. CD3 and CD8 density does not correlate with clinicopathological factors or p16/p53 expression, while CD57 and CD4 higher density is associated with the absence of distant metastases (p=0.03 and 0.07, respectively). Higher CD20 and S100 density is associated with lower T stage (p=0.04 and 0.03, respectively). PD-L1 expression is higher in CD3, CD8, and CD163 infiltrated tumors and in histologically more aggressive tumors. Response to neoadjuvant chemotherapy is better in highly CD3 infiltrated tumors and in tumors with less intraepithelial macrophages.

**Conclusion:**

Rich T-lympocytic and dendritic cell response is a good prognostic factor in SCCHN, whereas tumors expressing CTLA4 show poor prognosis. PDL1 expression does not affect prognosis, but it is expressed in histologically more aggressive tumors and in T-cells rich tumors. Response to induction chemotherapy is better in tumors less infiltrated by macrophages and mostly infiltrated by T cells.

## INTRODUCTION

Host's immune system influences cancer development so strong that the evaluation of local and systemic immunological markers has been shown to be a prognostic factor superior even of the TNM staging system [1]. Tumor-infiltrating immune cells have been identified as prognostic factors in lung [2], colorectal [3] and breast cancer [4] with different cell density, localization and type to be implicated. In line with these findings, we recently showed that a histologically rich lymphocytic host response, is a good prognostic factor in head-and-neck squamous cell carcinoma (SCCHN) both in surgical excision specimens but also in biopsy specimens [5, 6]. Furthermore, we found that it is a factor predictive of better response to induction chemotherapy (IC) [6].

At the same time, it is known that cancer can evade immune system influence through many mechanisms. Of these, two immunosuppressive pathways have gained great interest as they can be therapeutically targeted: the pathway of cytotoxic T lymphocyte antigen 4 (CTLA4), a molecule expressed by T cells inhibiting their function, and the programmed cell death 1 ligand 1 (PD-L1 or B7H1) pathway used by tumor cells to inhibit the antitumoral immune response.

However, the exact interaction between immune and anti-immune factors has not been studied in SCCHN, neither their prognostic or predictive role. In the current study, thus, we define the prognostic influence of the immune microenvironment in SCCHN by using markers for all major immune cells, namely B cells, T cells, cytotoxic T cells, NK cells, macrophages, dendritic cells and also immune checkpoints (Table 1), as these markers have been revealed of prognostic significance in various forms of cancer [2, 3, 7–19]. Furthermore, we reveal significant correlations between these different immune cells and clinicopathological parameters.

**Table 1:** Characteristics of immunohistochemical markers used

Marker	Principal role	Supplier	Clone	Dilution
**CD3**	T cell marker	Dako	F7,2,38	1/100
**CD8**	Cytotoxic T cell marker	Dako	C8/144B	1/100
**CD57**	NK and T cell marker	Leica	NK-1	ready to use
**Granzyme b**	Cytotoxic T cell granules	Novocastra	11F1	1/100
**CD4**	T-helper cell marker	Genemed	4B12	1/50
**CD20**	B cell marker	Dako	L26	1/200
**CD163**	Macrophage marker	Novocastra	10D6	1/200
**S100**	Dendritic cell marker	Dako	polyclonal	1/2500
**PD-L1**	Immune checkpoint	Cell Signaling	E1L3N	1/200
**CTLA-4**	Immune checkpoint	Origene	polyclonal	1/50
**CXCR4**	Chemokine receptor	Abcam	polyclonal	1/100
**P53**	Tumor-suppressor often altered in HNSCC	Dako	DO-7	1/50
**P16**	Often overexpressed in HPV-associated SCCHN	LabVision	16P07	1/50

Incubation time was 20 minutes for all markers. The automated Bond Leica system was used.

SCCHN Head-and-neck squamous cell carcinoma.

## RESULTS

### Clinicopathological and immunohistochemical characteristics

**Figure 1:**
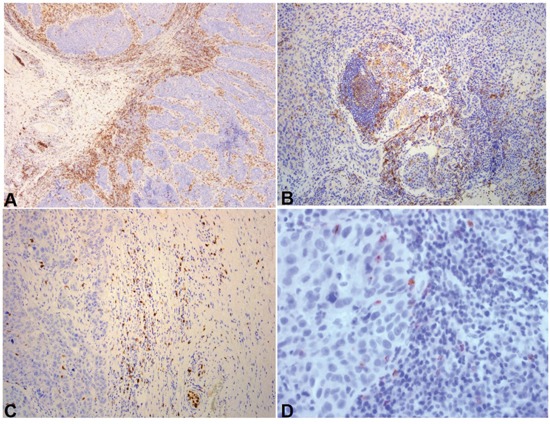
Representative immunohistochemical sections **A**. CD3 heavily infiltrated carcinoma. At the left of the image, front compartment. At the right, intense stromal infiltration. DAB x 100. **B**. CD4 moderately infiltrated carcinoma-stromal and intraepithelial compartment. DAB x 100. **C**. CD57 moderately infiltrated front compartment with sporadic cells in the stromal compartment. DAB x 100. **D**. Rare staining of lymphocytes for granzyme b in the intraepithelial compartment. DAB x 400.

Patients and tumors characteristics are presented in Table 2. Most patients were male (84.2%); the median age was 58.5 years at the time of diagnosis. Most tumors (94.8%) were diagnosed at an advanced stage. The median follow up was 24 months with 57.2% of the patients being alive at the time of the last follow up. Median overall and progression-free survival was 24 and 12 months respectively (Figure 1A, 1B). Of patients treated with IC, 42 (58.3 %) responded to chemotherapy.

**Table 2:** Clinical and histological characteristics

**Age**	
Range, median (years)	40-88, 58.5
**Sex**	
Female	24 (15.8%)
Male	128 (84.2%)
**Tumor location**	
Oropharynx	67 (44.1%)
Hypopharynx	49 (32.2%)
Larynx	36 (23.7%)
**Tumor status**	
T1/T2	38 (25%)
T3/T4	114 (75%)
**Nodal status**	
N0	29 (19.1%)
N1	23 (15.1%)
N2	73 (48%)
N3	27 (17.8%)
**Distant metastasis**	
M0	118 (77.6%)
M1	34 (22.4%)
**Stage**	
I	4 (2.6%)
II	4 (2.6%)
III	32 (21.1%)
IV	112 (73.7%)
**Status**	
Dead	65 (42.8%)
Alive	87 (57.2%)
**Follow up** (range, median in months)	3-84, 24
**Overall survival** (range, median in months)	3-84, 24
**Progression-free survival** (range, median in months)	3-72, 12
**Treatment**	
Radical excision	65 (42.8%)
Definite chemoradiotherapy	15 (9.8%)
Neoadjuvant chemotherapy (TPF)	72 (47.4%)
**Response to neoadjuvant chemotherapy (n=72)**	
Yes	42 (58.3%)
No	30 (41.7%)
**Histological subtype**	
Keratinising	110 (72.4%)
Non-keratinising	42 ( 27.6%)

**Figure 2:**
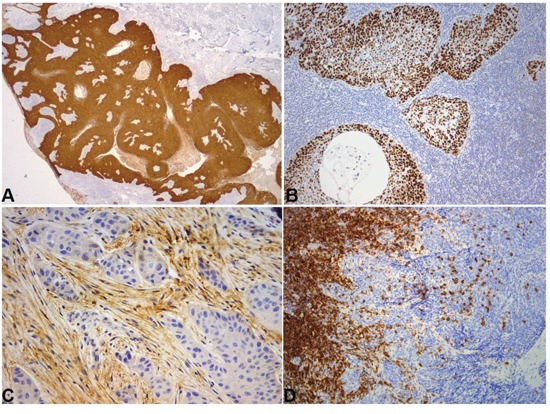
Representative immunohistochemical sections **A**. P16 strong cytoplasmic and nuclear expression. DAB x 25. **B**. P53 strong nuclear overexpression by all tumor cells. DAB x 100. **C**. CXCR4 expression by stromal cells was associated with a worse pattern of invasion. DAB x 200. **D**. CD20 heavily infiltrated frontal compartment. Moderately infiltrated intraepithelial compartment. DAB x 100.

**Figure 3:**
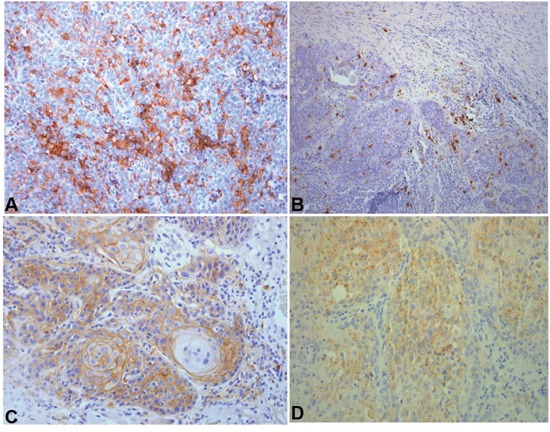
Representative immunohistochemical sections **A**. Tumors highly infiltrated by CD163 positive macrophages did not respond to induction chemotherapy. DAB x 200. **B**. S100 moderately infiltrated carcinoma. DAB x 100. **C**. Strong PD-L1 expression. DAB x 200. **D**. CTLA4 tumor cells expression. DAB x200. *DAB: 3,3′ diaminobenzidine*.

Immunohistochemical results are presented in Table 3. Representative images are shown in Figures 1–3.

**Table 3:** Immune-cell markers distribution

	Low (n,%)	High (n,%)
**CD3 sum**	68 (44.7)	84 (55.3)
CD3 intraepithelial	68 (44.7)	84 (55.3)
CD3 stromal	65 (42.7)	87 (57.3)
CD3 front	25 (38.5)	40 (61.5)
**CD8 sum**	64 (42.1)	88 (57.9)
CD8 intraepithelial	75(49.3)	77 (50.7)
CD8 stromal	51 (33.5)	101 (66.5)
CD8 front	11 (16.9)	54 (83.1)
**CD4 sum**	123 (81)	29 (19)
CD4 intraepithelial	150 (98.7)	2 (1.3)
CD4 stromal	129 (84.9)	23 (15.1)
CD4 front	60 (92.3)	5 (7.7)
**CD57 sum**	53 (34.9)	99 (65.1)
CD57 intraepithelial	108 (71)	44 (29)
CD57 stromal	81 (53.3)	71 (46.7)
CD57 front	33 (50.7)	32 (49.3)
**CD20 sum**	45 (29.4)	107 (70.8)
CD20 intraepithelial	137 (90.1)	15 (9.9)
CD20 stromal	72 (47.3)	80 (52.7)
CD20 front	22 (33.8)	43 (66.1)
**Granzyme b sum**	97 (63.9)	55 (36.1)
Granzyme b intraepithelial	134 (88.1)	18 (11.9)
Granzyme b stromal	114 (74.8)	38 (25.2)
Granzyme b front	53 (81.5)	12 (18.5)
**CD163 sum**	59 (38.8)	93 (61.2)
CD163 intraepithelial	88 (58.3)	64 (41.7)
CD163 stromal	42 (27.4)	110 (72.6)
CD163 front	22 (33.8)	43 (66.1)
**S100 sum**	50 (32.8)	102 (67.2)
S100 intraepithelial	52 (34.2)	100 (65.8)
S100 stromal	120 (78.9)	32 (21.1)
S100 front	59 (90.7)	6 (9.3)
**PDL1**	104 (68.4)	48 (31.6)
**CTLA4** tumor	124 (81.6)	28 (18.4)
CTLA4 lymphocytes	124 (81.6)	28 (18.4)
**CXCR4** tumor	79 (64.7)	43 (35.3)
CXCR4 stroma	96 (78.7)	26 (21.3)
CXCR4 lymphocytes	76 (62.3)	46 (37.7)
**P53 expression**		
Overexpression	93 (61.2%)	
Negative	36 (23.7%)	
Normal expression	23 (15.1%)	
**P16 expression**		
Positive	28 (18.4%)	
Negative	124 (81.6%)	

### Association of immune markers (Table 4) and other factors with OS and PFS

**Figure 4:**
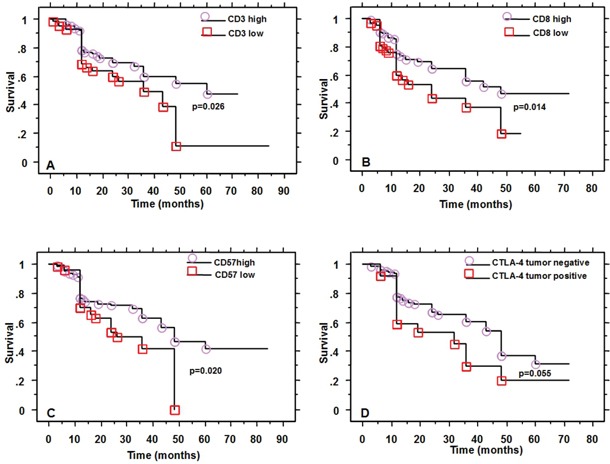
Survival of patients according to CD3, CD8, CD57 and CTLA4 expression

High density of CD3, CD8 and CD57 cells was associated with better OS and PFS (Figure 4). For CD3 density, the prognostic impact was stronger for the stromal compartment (p=0.007) than for the front (p=0.07) or the intraepithelial (p=0.06) compartment, whereas for CD8 and CD57 the intraepithelial (p=0.03 and 0.01, respectively) and the front compartment (p=0.03 and 0.0005, respectively) influenced prognosis stronger than the stromal one (p=0.1 and 0.4, respectively).

**Table 4:** Correlation of immune-cell markers density with overall (OS) and progression-free (PFS) survival (Mentel-Cox analysis)

	OS (months)	P	PFS (months)	P
**CD3**				
low	36	**0.02**	25	**0.04**
high	62		48	
**CD8**				
low	35	**0.01**	24	**0.01**
high	48		48	
**CD57**				
low	26	**0.02**	25	**0.04**
high	48		48	
**Granzyme b**				
low	48	0.3	35	0.3
high	44		44	
**CD4**				
low	48	0.6	35	0.4
high	44		35	
**CD20**				
low	44	0.3	36	0.3
high	48		36	
**CD163**				
low	44	0.4	35	0.2
high	35		35	
**S100**				
low	44	0.4	24	0.1
high	48		42	
**PDL1**				
low	35	0.4	36	0.6
high	60		36	
**CTLA4**-lymphocytes				
negative	44	0.4	35	0.4
positive	44		35	
**CTLA4**-tumor cells				
low	48	**0.05**	42	**0.05**
high	32		19	

Density of granzyme b, CD20, CD163 and S100 positive cells showed no association with survival. However, when seen by compartment, S100 higher density in stromal compartment did associate with better prognosis (p=0.07 for OS and 0.03 for PFS).

CTLA4 expression by tumor cells was associated with worse OS and PFS (p=0.05, Figure 4).

PDL1, CTLA4 lymphocytic expression, and CXCR4 expression were not associated with prognosis.

P16 and p53 expression was not associated with prognosis (log-rank p=0.26 and 0.81 respectively).

In terms of prognosis, supraglottic laryngeal cases (10 cases) were merged with pharyngeal cases as their treatment is similar. No association with prognosis was found (log-rank p=0.5 and 0.6, for OS and PFS, respectively).

### Association of immune markers with pathological features

The lymphocytic response as estimated by the BG-risk model corresponds to CD3 cells (p=0.001), CD8 cells (p=0.0009), CD57 cells (p=0.01) as well as B-lymphocytes (p=0.0001). The lymphocytes represented in the hematoxylin/eosin sections are not CD4 (p=0.4) or granzyme b (p=0.4) positive cells.

PDL1 expression showed a tendency (p=0.09) to be higher in tumors rich in lymphocytes and lower (p=0.06) in less aggressive tumors as estimated by the BG-score.

CTLA4 expression by lymphocytes was higher in tumors with rich lymphocytic response (p=0.002) and histologically (BG score) less aggressive (p=0.0009).

CXCR4 expression by stromal cells showed a tendency in associating with a worse pattern of invasion (p=0.06) and a worse BG-risk score (p=0.07).

### Association of immune markers with clinical features

CD3 and CD8 density did not show statistically significant correlation with T, N or M status or tumor localization.

The absence of distant metastases was associated with higher CD57 (p=0.03) and CD4 (p=0.07) density.

Lower T stage was associated with higher CD20 density (p=0.04) and higher S100 density (p=0.03).

Lower N stage was marginally associated with lower CTLA4 expression (p=0.09), while higher N stage was associated with higher CXCR4 lymphocytic expression (p=0.04).

Certain markers were associated with tumor localization: CD57 higher density was seen in oropharyngeal tumors (p=0.0007), CD4 lower density was seen in laryngeal tumors (p=0.03) and lower CTLA4 tumor cell expression in laryngeal carcinomas (p=0.04).

### Association between immune markers

PDL1 higher expression was associated with higher CD3 (p=0.08), CD8 (p=0.03), and CD163 (p=0.002) density; it was not associated with the rest of the immune markers. CTLA4 tumor expression was not associated with any other of the immune markers.

CTLA4 lymphocytic expression was associated with CD3 (p=0.02), CD8 (p=0.003), CD4 (p=0.007) and CD20 (p=0.05) density; it was not associated with the rest of the immune markers.

CXCR4 higher expression by lymphocytes showed a trend in associating with the density of CD3 lymphocytes (p=0.08).

Except for a trend for p16 positive tumors to also express CXCR4 (p=0.07), no association was found in any of the immune markers under investigation and p16 or p53 expression.

### Response to induction chemotherapy

Despite good response to IC was associated with a rich lymphocytic response (p=0.0001), as estimated in hematoxylin/eosin sections, and this lymphocytic response was correlated with CD3, CD8 and CD20 density (p= 0.01, 0.01 and 0.0009 respectively), good response to IC did not correlate with the immune markers studied, except for a strong trend (p=0.07) for CD3 density. Furthermore, CD163 lower intraepithelial density was associated with better response to IC.

## DISCUSSION

In this study, head-and-neck cancer patients whose tumors were densely infiltrated by T cells, cytotoxic T cells, CD57 positive cells or stromal dendritic cells had a better prognosis compared with patients whose tumors were poorly infiltrated. CD4 lymphocytes, granzyme B positivity, and macrophages did not affect prognosis. Interestingly, CD3 and CD8 infiltration did not correlate with any other clinical or histological factor, namely localization, T, N or M stage or histological subtypes, pattern of invasion or p16 or p53 expression. This implies that CD3 and CD8 immune response is largely a constitutive characteristic, independent of tumor's known clinicohistological features. By contrast, CD57 higher density was seen in oropharyngeal and in non-metastasized tumors. These could explain its positive prognostic role. CD4 higher density, despite not affecting prognosis, was associated with the absence of distant metastases. The positive prognostic role of T cells found here is consistent with their function, as cytotoxic T lymphocytes (CTL) kill tumor cells by stable contacts with them, through which they deliver them cytotoxic, perforin and granzyme-containing granules [20]. CD4 T lymphocytes primed by dendritic cells support CTL response and provide help to other cytotoxic cells of the innate and adaptive cells, such as NK cells or macrophages [21].

CD3 or CD8 infiltration has been studied separately or, rarer together, as prognostic factor in smaller series of SCCHN with various results as they have been revealed either as good prognostic factors [8, 15, 22–24], or they showed no association with outcome [11, 19, 25–28]. Given the differences found in relation to the tumor compartment, we consider possible that the discrepancies reported by the various studies originate from different ways of estimating immune markers. Recently in a series of 161 SCCHN treated by surgery and postoperative CRT, CD8 but not CD3 was a positive prognostic factor as evaluated in excision specimen [13], while in 101 biopsies of SCCHN treated with definitive CRT, CD3 and CD8 were both revealed as good prognostic factors [12].

CD3 higher density was associated with higher CD20 density but also CD163 density, revealing a relationship between T-lymphocytic reaction and B-lymphocytes and macrophages. Experimental data on SCCs suggest that B-cells promote tumor growth especially in the dysplasia state and that anti-CD20 monoclonal antibodies increase responsiveness in chemotherapy [9]. This latter effect requires macrophages and CD8 T cells action [9]. By contrast, in pulmonary squamous cell carcinomas, high CD10+/low CD20+ ratio was the only prognostic factor, showing actually a negative impact in survival [2]. In the current study, CD20 higher density was associated with a lower T stage, but not with prognosis. Similarly, chemoresponsiveness was not affected by CD20 density. These data show that B lymphocytes are a bystander of the T cytotoxic response, mostly important in earlier stage of the disease.

The impact of dendritic cells in SCCHN has been rarely reported in SCCHNs. In a study of 72 laryngeal carcinomas, they were revealed as positive prognostic factors [18]. Capture and presentation of antigens by DCs is a critical process in generating effective CTLs [21]. In line with this, we show that tumors rich in stromal dendritic cells have better prognosis and that higher S100 density is associated with a lower T stage.

Tumor-associated macrophages (TAMs), can either kill tumor cells or they can act as tumor promoters. Rich infiltration by TAMs is a poor prognostic factor in most tumors, like breast, urogential, gastric, ovarian, oral and thyroid or prostate tumors, but good for others, like colorectal cancer [29]. In lung cancer, correlation of TAMs with outcome depends on TAMs subsets and intratumoral distribution, as M2 type (CD163+) and stromal TAMs are correlated with poor outcome, while M1 type (HLA-DR+) and intraepithelial TAMs are correlated with favorable outcome [30]. In the present study, M2 macrophages did not affect prognosis but lower intraepithelial M2 macrophages did associate with better response to induction chemotherapy. These differences could reflect the different ways of evaluation or the differences between the various tumor types.

Except for poor T-lymphocytic response, the expression of CTLA4 by tumor cells also designated a poorer prognosis. This molecule is expressed in T-cells surface inhibiting their activation; one of the first immune checkpoint inhibitors approved by US Food and Drug Administration (FDA) was a monoclonal antibody that blocks CTLA4 and induced sustained antitumour responses [31]. However, the expression of CTLA4 by tumor cells has not been sufficiently studied. In a study of 81 lung carcinomas [17], 46.9% of the tumors overexpressed CTLA4; this showed a trend (p=0.078) in associating with better survival. By contrast, in 60 breast cancer patients [32], higher levels of tumoral CTLA4 were associated with advanced clinical stage. The adverse prognostic role of tumoral CTLA4 in SCCHN is herein for the first time reported. CTLA4 expression was seen mostly in non-laryngeal tumors, while no histological features predicted CTLA4 expression. Similarly, its expression by tumor cells, in contrast to that of PD-L1, was not influenced by the type of immune cells infiltration, showing that it is not an inflammation-association feature.

PD-L1 expression is increased in tumors providing them protection by reducing the activity of PD-1 expressing CD4 and CD8 T cells; monoclonal antibodies against PD-L1 have shown considerable results in melanoma, lung and renal cancer with clinical trials expanded in a variety of tumors including SCCHN [33]. In SCCHN, there are few studies mostly in tumors of oropharyngeal origin exploring PD-L1 expression, which varies from 46.4% to 100% of tumors studied, with contradictory findings regarding prognosis as seen in other solid tumors too [33]. These confounding findings probably result from the different clones of PD-L1 antibodies used and the detection methods, as well as the different ways of evaluation and cut-off points, the timing of the biopsy and the origin of the tissue [34–36] or even the section chosen, as PD-L1 can be expressed not uniformly but mostly at sites of immune cell infiltration [37]. Most of the SCCHN studies used a limited number of tumors. One of the biggest series concerned 305 oral carcinomas studied by tissue microarrays, and despite no PD-L1 association with survival was seen, when the study was limited to male smokers, an adverse prognosis was seen with PD-L1 expression [16]. In 55 carcinomas of the oral cavity, PD-L1 expression was inversely associated with CD8 density [27], in contrast to our results. PD-L1 has not been investigated with the rest of the immune markers or with detailed clinicohistological characteristics in SCCHN. In the current study, despite not associated with survival, PD-L1 was found to be expressed by histologically more aggressive tumors, specifically those with a more aggressive pattern of invasion. This is in agreement with recent studies in lung adenocarcinoma and SCCHN, which showed that tumors with epithelial to mesenchymal transition overexpress PD-L1 [38, 39]. Moreover, it was found mostly in tumors with higher lymphocytic response as estimated in hematoxylin/eosin sections but also by CD3 and CD8 staining, and not CD20 density, showing that tumors already rich in T-cells will probably benefit the most from such an immunotherapy.

CXCR4 is a chemokine receptor specific for CXCL12. It has been shown to promote angiogenesis, invasion and metastasis by leading tumor cells to tissues that release CXCL12 [40]. In line with this role, we found that its expression by stromal cells is associated with a worse pattern of invasion, while its expression by lymphocytes associated with more advanced N stage. Similarly, in 47 tongue SCC, its expression was associated with a more advanced N stage [10].

We have recently shown that the response to neoadjuvant chemotherapy was better in tumors heavily infiltrated by lymphocytes and in non-oropharyngeal tumors [6]. In this study, the response to TPF induction chemotherapy was marginally associated with a higher CD3 density and strongly associated with lower intraepithelial macrophages. As such, the inflammatory response estimated in hematoxylin/eosin sections is superior in predicting chemoresponsiveness than the isolated immune markers. It is probably the combined action of inflammatory cells that promotes this response. Also, the negative role of intraepithelial macrophages in response to TPF chemotherapy is for the first time demonstrated. Similar results have been found in esophageal cancer treated by neoadjuvant chemotherapy [41].

In conclusion, SCCHN highly infiltrated by T-cells and dendritic cells show better prognosis, whereas tumors expressing CTLA4 show poor prognosis. T-lymphocytic response is not associated with other clinical or histological features. PD-L1 expression does not affect prognosis, but it is expressed in histologically more aggressive tumors and in T-cell rich tumors. Response to induction chemotherapy is better in tumors less infiltrated by macrophages and mostly infiltrated by T cells, however, estimation of lymphocytic response by histological means, is a superior predictive factor of response to chemotherapy.

## PATIENTS AND METHODS

### Study population

One hundred and fifty two (152) consecutive patients diagnosed with SCCHN eligible for the following criteria were included in the study: (1) treatment with curative intent (2) a histological diagnosis of squamous cell carcinoma of the conventional type, excluding cases of spindle cell, verrucous, adenosquamous, basaloid, papillary, undifferentiated SCC (3) no prior treatment or another primary tumor and (4) a minimum follow up of 12 months or to death.

Sixty five (65) of the patients were surgically treated-radical surgery including resection of the primary tumor and neck dissection of regional lymph nodes-with or without (8 patients) adjuvant (chemoradiation) treatment, whereas 15 patients were treated with definitive chemoradiotherapy. Seventy two (72) of the patients were included in the therapeutic protocol of induction chemotherapy with docetaxel, cisplatin and 5-fluorouracil (TPF) followed by excision or chemoradiotherapy; for this group clinical and endoscopic evaluation of the response to induction chemotherapy were performed [6]. The response to IC was estimated from the last clinical and endoscopic evaluation based on the Response Evaluation Criteria in Solid Tumors (RECIST 1.1).

One hundred twenty two (122) patients were tested for HPV infection at the time of diagnosis. Four (4) were positive, so this was a HPV-negative cohort.

Local ethics committee approved the study (DC-2015-2489).

### Histopathological evaluation

All available slides were evaluated. Pathologists were blind regarding patients' status, response to chemotherapy or immunohistochemical results during this evaluation.

SCCs were divided into keratinizing (K-SCC) and non-keratinizing (NK-SCC) types, as previously suggested [42]. The Brandwein-Gensler (BG) histologic risk assessment model [43, 44] was used as a histoprognostic system that estimates (1) the worst pattern of invasion (WPOI), (2) the lymphocytic host response (LHR) and (3) the perineural invasion (PI).

Other histological features were also recorded: the absence or presence of large areas of *necrosis*, and the absence or presence of a marked *fibroblastic reaction*.

### Immunohistochemical analysis

Immunohistochemistry is a technique widely accepted as a means to study the various immune cells present in the tumor microenvironment as it offers the visualization of the cells in terms of quantity but also distribution. It has been previously used to evaluate microenvironment of various tumor types [2, 3, 7, 8, 12, 13].

Four-μm thick full sections were used for immunohistochemistry which was performed using an automated staining system (Leica Biosystems, Newcastle Upon Tyne, UK). In cases of neoadjuvant chemotherapy, pretreatment biopsies were used. Positive immunoreactions were visualized using 3,3′-diaminobenzidine as the chromogenic substrate. Primary antibodies and technical details are given in Table 1.

Regarding scoring for immune markers, cell infiltration of tumors was assessed by semiquantitative evaluation as previously described [3]: the density of positive cells was scored as 1: no or sporadic cells, 2: moderate number, 3: abundant occurrence and, 4: highly abundant. For granzyme b and CD4, this was modified, as staining was rarer, so the score 0 for completely negative and 1 for sporadic staining were introduced. In each tumor three compartments were evaluated: the intraepithelial compartment (cells within tumour cell nests), the stroma (cells within the intratumoural stroma) and the tumour periphery (cells localised in tumour periphery). In biopsy specimens, only the two components, that of the stroma and the intra-epithelial, were scored as the tumor front was not always present. In all cases, all available fields of the under examination section were examined. The total score was determined as the sum of the separate scores from the three (excision) or the two (biopsy) tumour compartments. The median value was used as a cutoff point in order to classify tumors into two groups: low or high expression. Similarly, the median value for each compartment was used to classify tumors into two groups, of low or high density, into the intraepithelial, the stromal and the peripheral compartment.

PD-L1 tumor cell staining was evaluated based on the proportion and the intensity of membranous and/or cytoplasmic staining. As no standard evaluation has been described so far, the following system was used [14]. Staining intensity: no staining; 1, weak staining; 2, moderate staining; and 3, strong staining. Frequency of stained cells: 1, less than 1% staining; 2, staining in 1% to 10%; 3, staining in 10% to 33.3%; 4, staining in 33.3% to 66.6%; and 5, more than 66.6%. The final score ranging from 0 to 8 was the sum of the 2 scores. For statistical purposes, a dichotomous classification was determined, taking as threshold the median of the score.

CTLA4 showed a granular cytoplasmic staining; it was recorded separately in tumor cells and lymphocytes. It was classified in a binary manner as either absent or present for lymphocytes and as positive or negative for the tumor with a cut-off value of 5% in this latter case.

A subset of tumors (n=122) was tested for CXCR4 expression. CXCR4 was recorded in tumor cells, lymphocytes and stromal fibroblasts. It was classified in a binary manner as either absent or present.

Also, p16 and p53 were used as an adjunct to the characterization of the tumors, the first as a surrogate marker of HPV infection [45, 46] and the second as a major tumor-suppressor altered in SCCHN of tobacco users [47]. For p16, cases were classified in a binary manner as either positive (nuclear/cytoplasming diffuse staining of more than 75% of cells) or negative [5, 6]. P53 staining was nuclear. Three patterns of p53 expression were recognized: (a) overexpression (strong nuclear staining by at least 50% of the cells) (b) negative (c) normal p53 expression when a weak expression of few tumor cells (weak expression by no more than 49%) was found [5, 6].

### Statistical analysis

Data were analyzed using the StatView software (Abacus Concepts, Berckley Ca, USA). Relationship between two groups was investigated using chi-square or Fisher's exact test for categorical data. Survival probability was estimated by Kaplan–Meier analysis. For all analyses, statistical significance was indicated at a *p* value of < 0.05.
